# Causality Is an Effect, II

**DOI:** 10.3390/e23060682

**Published:** 2021-05-28

**Authors:** Lawrence S. Schulman

**Affiliations:** Physics Department, Clarkson University, Potsdam, NY 13699-5820, USA; schulman@clarkson.edu

**Keywords:** causality, arrow of time, entropy increase, quantum generalization, Gaussian wave packets

## Abstract

Causality follows the thermodynamic arrow of time, where the latter is defined by the direction of entropy increase. After a brief review of an earlier version of this article, rooted in classical mechanics, we give a quantum generalization of the results. The quantum proofs are limited to a gas of Gaussian wave packets.

## 1. Introduction

The history of multiple time boundary conditions goes back—as far as I know—to Schottky [1,2], who, in 1921, considered a single slice of time inadequate for prediction or retrodiction (see Appendix A). There was later work of Watanabe [3] concerned with prediction and retrodiction. Then, Schulman [4] uses this as a conceptual way to eliminate “initial conditions” prejudice from Gold’s [5] rationale for the arrow of time and Wheeler [6,7] discusses two time boundary conditions. Gell–Mann and Hartle also contributed to this subject [8] and include a review of some previous work. Finally, Aharonov et al. [9,10] proposed that this could solve the measurement problem of quantum mechanics, although this is disputed [11]. See also Appendix B.

This formalism has also allowed me to come to a conclusion: effect follows cause in the direction of entropy increase. This result is not unanticipated; most likely everything having to do with arrows of time has been anticipated. What is unusual, however, is the ability to prove the thesis mathematically.

The proof given [12] only applies to classical mechanics. The present work extends it to quantum mechanics. Moreover, since the previous paper was buried in conference proceedings, and the present work uses similar arguments, I will repeat some parts of that proof. Note though that the present work is extremely limited: it applies to particles in a gas with Gaussian wave functions. I think it should apply more generally, but that is not what I prove.

A remark is in order on the arrow of time. Often the sequence of cause and effect is taken as primary [13], but in the present context this is shown not to hold. Our argument depends on two conditions: the nature of perturbation and two-time boundary conditions. Both will be elaborated on. A future condition on quantum problems may make measurement deterministic but is hollow in that it does not lead (to my knowledge) to testable claims. There is the possibility of advanced effects ([6,14]) but so far, this has not proved measurable.

## 2. Perturbation

A “cause” can be a change (or intervention, in the dynamics or in the initial conditions) or it can be a precursor (“pre” in the usual meaning of the term). In a change, you can compare the outcome of two similar but not identical “causes.” The case of “precursor” is different. You can say one thing leads to another, A is a precursor of B, but you only consider A, not some variant, say A${}^{\prime }$. I will adopt the meaning “change.” This allows study of “effects”, whereas I do not know how to examine ordinary time evolution. One should bear in mind, however, that in ordinary language both meanings of “cause” can be used. This is also discussed in Appendix B of [12], where a rationale for this choice is given.

## 3. Review of Previous Work

The argument for causality to follow the direction of entropy increase depends on the dynamics. The principle assumption on the dynamics is that there is a relaxation time. Call it τ (this is aside from the cosmological assumptions, discussed below). Let the dynamics be ϕ, so that from time *t* to t+1 a subset of ϵ of the phase space goes from ϵ to ϕϵ. Then, by giving two times separated by more than 2τ and low entropy in the beginning and end, one gets increase, then constant, then decline of the entropy. Thus, the set of points satisfying both boundary conditions ($\epsilon_{0}$ at 0 and $\epsilon_{T}$ at *T*) at time 0 is

(1) $$\epsilon =\epsilon_{0}\cap \phi^{-T}\epsilon_{T}\;.$$

**Figure 1:**
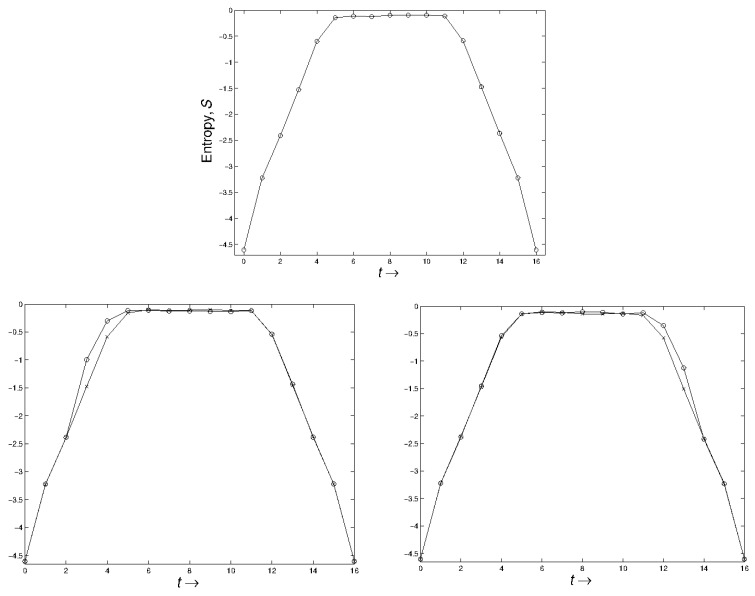
The entropy as a function of time for the “cat map” is plotted. In all three figures the unperturbed entropy is shown. In the second figure the perturbation (with circles around values) takes place at time t=3, while in the third figure the perturbation is at time t=13. In the latter two cases the change in macroscopic behavior is *subsequent* in the sense of increasing entropy. (The graphs are inverted from the usual definition of entropy.)

Note that both beginning and end are forced to have low entropy (by definition, assuming $\epsilon_{0}$ and $\epsilon_{T}$ are small). This is illustrated for the “cat map” in the first graph of Figure 1. See Appendix C for information on the “cat map” and on our procedure.

Then, we do something different! Two times for perturbation are contemplated: the first during the rising of the entropy, the second during its fall. By detailed calculation (not reproduced here, but see below, Section 5) it is found that causality, the macroscopic change in behavior, follows the direction of entropy increase, that is in the falling entropy case, the macroscopic behavior changes earlier in the time parameter (which is neutral, i.e., has two different arrows and in between doesn’t have an arrow).

Intuitively, this is simple. Let the perturbation be at $t_{0}$ and $0<t_{0}<\tau <T-\tau <T$. Between 0 and $t_{0}$ (before the perturbation) both perturbed and unperturbed have the same boundary conditions, hence their macroscopic behavior is similar (but the microscopic behavior is, in general, different). After the perturbation there is no constraint, i.e., no effective boundary condition: both systems go to equilibrium, from which there is adequate time (recall T>2τ) to reach (whether perturbed or not) a specific region within the unit square. There was however a perturbation and there are different microscopic *and* macroscopic paths. Similarly, for $T-\tau <t_{0}<T$ the boundary conditions at $\epsilon_{T}$ and $t_{0}$ (just after the perturbation in the neutral parameter *t*) are the same for perturbed and unperturbed systems, hence the macroscopic behavior is the same. However, they both go (in (−t)) to equilibrium; hence, the two systems (perturbed and unperturbed) have different macroscopic paths (N.b., distinguish carefully the words “macroscopic” and “microscopic.”).

This is illustrated in the case of the cat map, both for $0<t_{0}<\tau$ and $T-\tau <t_{0}<T$. See Figure 1, second and third images.

## 4. Quantum Version

The first step is to show that with low entropy conditions at both (distantly separated) times, the entropy increases in between. To calculate entropy in subspaces of Hilbert space presence or absence in that subspace should be defined and numbers of states counted. This leads to having regions of 6-dimensional x-p space—to mimic the classical space—and that is possible. What we do is take a region of phase space and select a basis of states whose maximum value is in this region. Coherent states will do the job. For convenience, the value of the spread used to define those states might be the fixed point of [15], but that is not necessary. Finally the dimension of the Hilbert subspace will be the number of states in that region. What this means is that one can go back to the classical method of using (the logarithm of) volume in phase space as a measure of entropy.

Therefore, as was done previously, we take coarse grains that are regions of phase space—coordinates and momentum. The density matrix involves mainly the diagonal elements. Thus even for identical particles the separation of locale as well as separation of momenta makes the density matrix nearly diagonal. For more on this theme, see [16].

Even for identical particles this leads to cancellation. In one dimension the wave function for a pair of Gaussians is (not normalized)

(2) $$\begin{array}{c} \exp\left\{-\left[\frac{{\left(x_{1}-x_{\alpha }\right)}^{2}}{4\sigma_{\alpha }^{2}}+\frac{{\left(x_{2}-x_{\beta }\right)}^{2}}{4\sigma_{\beta }^{2}}\right]+ik_{\alpha }x_{1}+ik_{\beta }x_{2}\right\}\\ \;\pm \exp\left\{-\left[\frac{{\left(x_{2}-x_{\alpha }\right)}^{2}}{4\sigma_{\alpha }^{2}}+\frac{{\left(x_{1}-x_{\beta }\right)}^{2}}{4\sigma_{\beta }^{2}}\right]+ik_{\alpha }x_{2}+ik_{\beta }x_{1}\right\}\;. \end{array}$$

(The variables are $x_{1}$ and $x_{2}$; all the others are constants.) The diagonal elements of the density matrix (calculated from Equation (2)) already show signs of cancellation, as follows:

(3) $$\begin{array}{ccc} \rho (x_{1},x_{2};x_{1},x_{2}) & = & \exp\left[-\frac{{\left(x_{1}-x_{\alpha }\right)}^{2}}{2\sigma_{\alpha }^{2}}-\frac{{\left(x_{2}-x_{\beta }\right)}^{2}}{2\sigma_{\beta }^{2}}\right]+\exp\left[-\frac{{\left(x_{2}-x_{\alpha }\right)}^{2}}{2\sigma_{\alpha }^{2}}-\frac{{\left(x_{1}-x_{\beta }\right)}^{2}}{2\sigma_{\beta }^{2}}\right]\\ \; & \; & \;+\exp\left[-\frac{{\left(x_{1}-x_{\alpha }\right)}^{2}}{4\sigma_{\alpha }^{2}}-\frac{{\left(x_{2}-x_{\beta }\right)}^{2}}{4\sigma_{\beta }^{2}}-\frac{{\left(x_{2}-x_{\alpha }\right)}^{2}}{4\sigma_{\alpha }^{2}}-\frac{{\left(x_{1}-x_{\beta }\right)}^{2}}{4\sigma_{\beta }^{2}}\right]\\ \; & \; & \;\;\;\times 2\left\{\;\begin{array}{c} \cos\\ i\sin\; \end{array}\right\}\left(k_{\alpha }-k_{\beta }\left[x_{1}-x_{2}\right]\right)\;. \end{array}$$

As is evident, if $x_{\alpha }$ is significantly different from $x_{\beta }$ or $k_{\alpha }$ from $k_{\beta }$, then there is already cancellation or rapid oscillation. With more particles the effect is stronger. This, by the way, is the reason that isolated systems can be analyzed without paying attention to symmetrization with respect to all identical particles in the universe.

To get low entropy at the beginning and the end confine the wave functions—at the beginning and end—to particular coarse grains. In between the two times considered, wave functions will spread. This sounds like the classical definitions, but once you have coarse grains the definitions are not all that different. However, to imitate [12] it is necessary that $\psi_{\mathrm{final}}=U_{T}\psi_{\mathrm{initial}}$, where $U_{T}$ is the propagator from time-0 (zero is the initial time) to time-*T* (*T* is the final time). This imposes a significant constraint on the wave function, in particular, the wave function, under $U_{T}$, should not spread. If spreading were to happen, the entire space available, not just the target in phase space, would be occupied and the entropy would not drop.

In a recent article [15], we found that Gaussians that scatter do not spread. (This was explored further in [17], but the principal application to Gaussians was done in [15].) The idea is that scattering provides localization. In [17], it is argued that wave functions become Gaussian (often) but that involves decoherence, which is human perception. Is everything a Gaussian? Obviously not; atomic wave functions can be complicated and even the hydrogen atom is a different function. Nevertheless, for the purpose of this demonstration a Gaussian will be adequate, at least for showing that *sometimes* causality is an effect.

The requirement that $\psi_{\mathrm{final}}=U_{T}\psi_{\mathrm{initial}}$*and* that both be confined to a (small) region of phase space (at t=0 and t=T) is severe. However, based on the results of [15], it can be done. It is possible that the entropy would not be strictly zero (due to the Gaussian’s never vanishing) but it can be made small. The same holds in momentum space.

At this point we are back in classical mechanics and the proof is straightforward. Since non-standard definitions are used in [12] we repeat the proof (now using standard definitions).

## 5. Classical Proof

We present a précis of what has been done in our previous work ([12]).

On the phase space Ω let μ be a measure and μ(Ω)=1. Let the dynamics be a measure-preserving map $\phi^{\left(t\right)}$ on Ω, with $\phi^{\left(t\right)}\left(\omega \right)$ the time-*t* image of an initial point ω∈Ω. The coarse graining of Ω, providing a notion of “macroscopic,” are sets with the following properties: $\left\{\Delta_{\alpha }\right\}$, α=1,…,G, with $\cup_{\alpha }\Delta_{\alpha }=\Omega$, $\Delta_{\alpha }\cap \Delta_{\beta }=⌀$ for α≠β. Let $\chi_{\alpha }$ be the characteristic function of $\Delta_{\alpha }$ and let $v_{\alpha }=\mu \left(\Delta_{\alpha }\right)=\int \chi_{\alpha }\left(\omega \right)d\omega$ (ω∈Ω). If *f* is a function on Ω, its coarse graining is defined as

(4) $$\hat{f}\left(\omega \right)\equiv \sum_{\alpha }\chi_{\alpha }\left(\omega \right){\hat{f}}_{\alpha }\;\;\mathrm{with}\;\;{\hat{f}}_{\alpha }\equiv \frac{\int d\omega \;\chi_{\alpha }\left(\omega \right)f\left(\omega \right)}{v_{\alpha }}\;.$$

Let the system’s distribution in Ω be described by a density function ρ(ω). The entropy to be used for studying irreversibility involves coarse graining and is defined as

(5) $$S\left(\rho \right)\equiv -\int_{\Omega }\hat{\rho }\log\hat{\rho }\;d\mu \;.$$

with $\hat{\rho }$ formed from ρ as in Equation (4). (In other notation, $S=-\int_{\Omega }\hat{\rho }\left(\omega \right)\log\hat{\rho }\left(\omega \right)\;d\omega$.) The *relative entropy* (Kullback–Leibler divergence), to which S(ρ) is related, was given in [12] with a different sign from the usual. Moreover, the illustration given in Figure 1 uses a different definition of entropy.

Turning to the system at hand, it is required to start (t=0) in a subset $\epsilon_{0}\subset \Omega$ and end (t=T) in a subset $\epsilon_{T}\subset \Omega$. (The fact that Gaussian wave functions necessarily do not vanish anywhere may lead to a small correction.) The points of Ω satisfying this two-time boundary condition are

(6) $$\epsilon =\epsilon_{0}\cap \phi^{\left(-T\right)}\left(\epsilon_{T}\right)\;.$$

In [12,18,19] it is argued that for chaotic dynamics and for sufficiently long times *T*, ϵ≠⌀. (Whether this carries over to quantum mechanics will be dealt with later.) We assume that there is a relaxation time τ, and that T≫τ. As a consequence

(7) $$\mu \left(\epsilon \right)∼\mu \left(\epsilon_{0}\right)\mu \left(\epsilon_{T}\right)\;.$$

(Recall that for mixing dynamics, $\phi^{\left(t\right)}$ satisfies $\lim_{t\to \infty }\mu \left(A\cap \phi^{\left(t\right)}\left(B\right)\right)=\mu \left(A\right)\mu \left(B\right)$. This is true for the t→∞ limit, but we assume that there is a time (τ) such that the decorrelation condition holds. Moreover, ϕ is measure preserving.) Under $\phi^{\left(t\right)}$, ϵ becomes

(8) $$\epsilon \left(t\right)=\phi^{\left(t\right)}\left(\epsilon_{0}\right)\cap \phi^{\left(t-T\right)}\left(\epsilon_{T}\right)\;.$$

To calculate the entropy, the density, which was $\rho \left(0\right)=\chi_{\epsilon }/\mu \left(\epsilon \right)$ at time-0, must be coarse grained. The important quantity for the entropy calculation is

(9) $$\rho_{\alpha }\left(t\right)=\frac{\mu \left(\Delta_{\alpha }\cap \epsilon \left(t\right)\right)}{\mu (\epsilon )}=\frac{\mu \left(\Delta_{\alpha }\cap \phi^{\left(t\right)}\left(\epsilon_{0}\right)\cap \phi^{\left(t-T\right)}\left(\epsilon_{T}\right)\right)}{\mu (\epsilon )}\;.$$

If T−t>τ then the following will hold

(10) $$\begin{array}{cc} \mu \left(\Delta_{\alpha }\cap \phi^{\left(t\right)}\left(\epsilon_{0}\right)\cap \phi^{\left(t-T\right)}\left(\epsilon_{T}\right)\right) & =\mu \left(\Delta_{\alpha }\cap \phi^{\left(t\right)}\left(\epsilon_{0}\right)\right)\mu \left(\phi^{\left(t-T\right)}\left(\epsilon_{T}\right)\right)\;,\\ \mu \left(\epsilon \right) & =\mu \left(\epsilon_{0}\right)\mu \left(\phi^{\left(-T\right)}\left(\epsilon_{T}\right)\right)\;. \end{array}$$

Using the measure-preserving property of $\phi^{\left(t\right)}$, the factors $\mu (\epsilon_{T})$ in both numerator and denominator of $\rho_{\alpha }$ cancel, leading to

(11) $$\rho_{\alpha }\left(t\right)=\frac{\mu \left(\Delta_{\alpha }\cap \phi^{\left(t\right)}\left(\epsilon_{0}\right)\right)}{\mu (\epsilon_{0})}\;.$$

This is precisely what one gets *without* future conditioning, so that all macroscopic quantities, and in particular the entropy, are indistinguishable from their unconditioned values.

Working backward from time-*T* one obtains an analogous result. Define a variable s≡T−t and set $\tilde{\epsilon }\left(s\right)\equiv \epsilon \left(T-s\right)$. Then

(12) $$\tilde{\epsilon }\left(s\right)=\phi^{\left(T-s\right)}\left(\epsilon_{0}\right)\cap \phi^{\left(-s\right)}\left(\epsilon_{T}\right)\;.$$

If *s* satisfies T−s>τ, then when the density associated with $\tilde{\epsilon }\left(s\right)$ is calculated, its dependence on $\epsilon_{0}$ will drop out. It follows that

(13) $$\rho_{\alpha }\left(s\right)=\frac{\mu \left(\phi^{\left(-s\right)}\left(\epsilon_{T}\right)\right)}{\mu (\epsilon_{T})}\;.$$

For a time-reversal invariant dynamics this will give the entropy the same time dependence coming back from *T* as going forward from 0. Even in the absence of T invariance, there should be roughly the same behavior because of the absence of any dissipative dynamics.

Now we turn to perturbations. Call the unperturbed system A. The microstates are in the set

(14) $$\epsilon_{A}=\epsilon_{0}\cap \phi^{\left(-T\right)}\left(\epsilon_{T}\right)$$

(formerly called ϵ). System B, the perturbed case, has an instantaneous transformation act on it at time-$t_{0}$. Call this transformation ξ. (This transformation is called ψ in [12]. The letter ξ is chosen to avoid confusion with the wave function.) It is not dissipative—the arrow does not arise from such an asymmetry. ξ is invertible and measure preserving. Successful solutions must go from $\epsilon_{0}$ to $\epsilon_{T}$ under the transformation $\phi^{\left(T-t_{0}\right)}\xi \phi^{\left(t_{0}\right)}$. The microstates for system B are therefore

(15) $$\epsilon_{B}=\epsilon_{0}\cap \phi^{\left(-t_{0}\right)}\xi^{-1}\phi^{\left(-T+t_{0}\right)}\left(\epsilon_{T}\right)$$

Again, there is the assumption that $\epsilon_{B}$ is not empty, which will be taken up for quantum mechanics in Section 6. Clearly, $\epsilon_{A}$ and $\epsilon_{B}$ are different—at *all* times. But as will now be shown, for mixing dynamics and for sufficiently large *T*, the following hold: (1) for $t_{0}$ close to 0, the only differences in macroscopic behavior between A and B are for $t>t_{0}$; (2) for $t_{0}$ close to *T*, the only differences in macroscopic behavior between A and B are for $t<t_{0}$. *The direction of causality follows the direction of entropy increase.*

The proof is nearly the same as before. Again we use a time τ such that the mixing decorrelation holds for time intervals longer than τ. First consider $t_{0}$ close to 0. The observable macroscopic quantities are the densities in grain-$\Delta_{\alpha }$, which are, for $t<t_{0}$,

(16) $$\begin{array}{c} \rho_{\alpha }^{A}\left(t\right)=\mu \left(\Delta_{\alpha }\cap \phi^{\left(t\right)}\left(\epsilon_{0}\right)\cap \phi^{\left(t-T\right)}\left(\epsilon_{T}\right)\right)/\mu \left(\epsilon_{A}\right)\;,\\ \rho_{\alpha }^{B}\left(t\right)=\mu \left(\Delta_{\alpha }\cap \phi^{\left(t\right)}\left(\epsilon_{0}\right)\cap \left[\phi^{\left(t-t_{0}\right)}\xi^{-1}\phi^{\left(t_{0}-T\right)}\right]\left(\epsilon_{T}\right)\right)/\mu \left(\epsilon_{B}\right)\;. \end{array}$$

As before, the mixing property, for T−t>τ, yields $\rho_{\alpha }^{A}\left(t\right)=\mu \left(\Delta_{\alpha }\cap \phi^{\left(t\right)}\left(\epsilon_{0}\right)\right)/\mu \left(\epsilon_{0}\right)$, which is the initial-value-only macroscopic time evolution. For $\rho_{\alpha }^{B}$, the only difference is to add a step, $\xi^{-1}$. But this step is as measure preserving as ϕ itself, and therefore, as before, $\frac{\left[\phi^{\left(t-t_{0}\right)}\xi^{-1}\phi^{\left(t_{0}-T\right)}\right]\left(\epsilon_{T}\right)}{\mu (\epsilon_{B})}=\frac{1}{\mu (\epsilon_{0})}$. Thus A and B have the same macrostates before $t_{0}$.

For $t>t_{0}$, $\rho_{\alpha }^{A}\left(t\right)$ continues its behavior as before. For $\rho_{\alpha }^{B}\left(t\right)$ things are different:

(17) $$\rho_{\alpha }^{B}\left(t\right)=\mu \left(\Delta_{\alpha }\cap \left[\phi^{\left(t-t_{0}\right)}\xi \phi^{\left(t_{0}\right)}\right]\left(\epsilon_{0}\right)\cap \phi^{\left(t-T\right)}\left(\epsilon_{T}\right)\right)/\mu \left(\epsilon_{B}\right)\;\;\;\;\;\left(t>t_{0}\right).$$

Now, I require T−t>τ. If this is satisfied the $\epsilon_{T}$ dependence drops out and

(18) $$\rho_{\alpha }^{B}\left(t\right)=\mu \left(\Delta_{\alpha }\cap \left[\phi^{\left(t-t_{0}\right)}\xi \phi^{\left(t_{0}\right)}\right]\left(\epsilon_{0}\right)\right)/\mu \left(\epsilon_{0}\right)\;.$$

This shows that the effect of ξ is the usual initial-conditions-only phenomenon.

If we repeat these arguments for *t* such that T−t is small, then just as we showed in Section 3, the effect of ξ will only be at times *t less than* $t_{0}$.

## 6. The Set $\epsilon_{0}\cap \phi^{-T}\epsilon_{T}$ Is Not Empty under Quantum Evolution

The assertion, the set $\epsilon_{0}\cap \phi^{-T}\epsilon_{T}$ is not empty under quantum evolution, is *sometimes* true. What has been shown is that Gaussian wave functions do not spread indefinitely, but rather are localized each time they scatter [15]. Thus, our arguments hold for a gas of particles that are Gaussians and occasionally scatter. As found in [15,17], wave functions overlap, but scattering usually takes place at distances on the order of ℓ= mean free path (for scattering).

What happens in more general situations is not known. One theory of the quantum measurement process ([19], namely, there is no such thing as a measurement) proposes that non-entanglement is usually the case. However, experiment has not yet ruled on this issue.

## 7. Conclusions

There are many defects to this quantum version of entropy increase determining the direction of causality. What is not clear is whether a quantum theory is at all needed. Causality as usually understood is a macroscopic phenomenon and not quantum mechanical. Nevertheless, we point out the deficiencies of the present work.

There is uncertainty over black holes. If one considers two time boundary conditions purely as a way to get rid of “initial conditions” prejudice, OK, but if there is any thought that the world is actually symmetric (approximately) then that too must be dealt with. Do black holes evaporate? Do they become white holes? Are there black holes at all? (See the blog by Cramer [20].) We will not deal with any of these questions and allow our results to depend on the answers.

Our arguments are phrased in terms of chaotic dynamics. Chaos is problematic for quantum mechanics. Our previous paper ([12]) deals also with harmonic oscillators (which are not chaotic) but has other limitations. For the argument above to be meaningful all that is really required is a relaxation time. It is plausible that this exists in quantum theory.

Although we have briefly mentioned identity of particles, that has not been seriously dealt with.

Finally, there is the requirement that $\psi_{\mathrm{final}}=U_{T}\psi_{\mathrm{initial}}$ (where $U_{T}$ is the evolution operator for time *T*) and that both be low entropy, confined to a region of phase space. In some versions of quantum mechanics this is a natural requirement, but if that is wrong, there are severe limitations on the applicability of these results (see Section 6).

We mention in passing that our understanding of the “destiny” of Aharonov et al. does not fix the results of measurements (the “destiny” being a future value of the wave function). This is because general wave functions allow many outcomes to a given experiment.

## Data Availability

Not Applicable.

## Appendix A. Justification of Schottky

The assertions of Schottky [1,2] were later justified in the context of Wheeler-Feynman (and predecessors) theory by Schulman [21]. The cited paper only shows the existence problem when two particles are involved. It is clear that if there are more, confusion reigns. This is not to say that the initial value problem by conventional electrodynamics (with the fields explicit) lacks a solution. But the field-free formulation presents further difficulties.

## Appendix B. More on History

This is a bare bones review of the history of two time or two state boundary conditions. In particular there is other work by Bopp [22], Craig [23], Hegerfeldt [24], Schulman [25] and many others on related subjects.

## Appendix C. The “Cat Map”

The “cat map” is a gas of *N* non-interacting particles, each obeying $x^{\prime }=x+y$, $y^{\prime }=x+2y$, both mod 1, and both *x* and *y* in the unit square. See [26]. The entropy is $S=-\sum p_{\alpha }\log p_{\alpha }$ with $p_{\alpha }=n_{\alpha }/N$ where $n_{\alpha }$ is the number of points in a box of a $\frac{1}{M}$-by-$\frac{1}{M}$ grid, $N=\sum n_{\alpha }$ (=500 in the figure) and *M* (=10) is an integer.

The name, due to Arnold, is derived from an image of a cat, lines in the unit square, that become highly distorted in a few time steps.

In the figures a few more than $NM^{2}$ points are started in the first region and only those which land in the final region are kept. This turns out to be symmetric and is a realization of the set ($\epsilon =\epsilon_{0}\cap \phi^{-T}\epsilon_{T}$ or versions involving ξ) in the analytic version.
